# The impact of direct admission to a catheterisation lab/CCU in patients with ST-elevation myocardial infarction on the delay to reperfusion and early risk of death: results of a systematic review including meta-analysis

**DOI:** 10.1186/s13049-014-0067-x

**Published:** 2014-11-25

**Authors:** Magnus Andersson Hagiwara, Anders Bremer, Andreas Claesson, Christer Axelsson, Gabriella Norberg, Johan Herlitz

**Affiliations:** School of Health Sciences, The Centre for Pre-hospital Research, University of Borås, SE-501 90 Borås, Sweden; Inst of Medicine, Department of Molecular and Clinical Medicine, Sahlgrenska University Hospital, SE-413 45 Göteborg, Sweden

**Keywords:** Emergency medicine, Myocardial ischemia and infarction, Intensive care

## Abstract

**Background:**

For each hour of delay from fist medical contact until reperfusion in ST-elevation myocardial infarction (STEMI) there is a 10% increase in risk of death and heart failure. The aim of this review is to describe the impact of the direct admission of patients with STEMI to a Catheterisation laboratory (cath lab) as compared with transport to the emergency department (ED) with regard to delays and outcome.

**Methods:**

Databases were searched for from April-June 2012 and updated January 2014: 1) Pubmed; 2) Embase; 3) Cochrane Library; 4) ProQuest Nursing and 5) Allied Health Sources. The search was restricted to studies in English, Swedish, Danish and Norwegian languages.

The intervention was a protocol-based clinical pre-hospital pathway and main outcome measurements were the delay to balloon inflation and hospital mortality.

**Results:**

Median delay from door to balloon was significantly shorter in the intervention group in all 5 studies reported. Difference in median delay varied between 16 minutes and 47 minutes.

In all 7 included studies the time from symptom onset or first medical contact to balloon time was significantly shorter in the intervention group. The difference in median delay varied between 15 minutes and 1 hour and 35 minutes. Only two studies described hospital mortality. When combined the risk of death was reduced by 37%.

**Conclusion:**

An overview of available studies of the impact of a protocol-based pre-hospital clinical pathway with direct admission to a cath lab as compared with the standard transport to the ED in ST-elevation AMI suggests the following. The delay to the start of revascularisation will be reduced. The clinical benefit is not clearly evidence based. However, the documented association between system delay and outcome defends the use of the pathway.

**Electronic supplementary material:**

The online version of this article (doi:10.1186/s13049-014-0067-x) contains supplementary material, which is available to authorized users.

## Introduction

During the last few decades, the treatment of patients with a presumed acute coronary syndrome (ACS) has focused on the concept of “time is muscle”. This concept has become particularly relevant with regard to patients with ST-segment-elevation myocardial infarction (STEMI) and reperfusion therapy [1,2].

It was recognised at an early stage that the pre-hospital initiation of therapy with thrombolysis was valuable with regard to reperfusion [3]. However, nowadays, a large number of patients with STEMI are offered reperfusion therapy with early angioplasty [1].

One way to speed up the process was to admit the patients directly to a coronary care unit (CCU) which, at a time of high thrombolysis use, clearly shortened the door-to-needle time in individual cases. Since the start of these individual experiences, a number of observational studies have attempted to explore the effect, on patients with STEMI, of direct admission to a catheterisation laboratory (cath lab) or CCU as compared with the traditional transport to the emergency department (ED). In these studies, the aim has been to address the effect on delay and possible effects on patient outcome. To our knowledge, there is no previous systematic review which has evaluated the effect of direct admission to a cath lab. Since clinical pathways of all kinds are resource consuming in relation to the pre-hospital organisation, we believe that patient benefits must be thoroughly evaluated in clinical pathways of all kinds.

The primary aim of this survey was to summarise the present knowledge based on the available literature on the impact of direct admission to a cath lab as compared with transport to the ED with regard to delays and outcome among patients with STEMI.

## Method

We have searched for randomised controlled trials (RCT) that compared the pre-hospital pathway with standard care. We have also included studies with weaker designs, such as prospective and retro-perspective observational studies, before and after studies and studies with a time series design. The plan for the studies that are included was to have two groups so that a comparison could be made.

As a quality assessment tool, the Sign 50 checklist [4] was used. This checklist is considered to be a satisfactory tool for assessing study characteristics [5]. To be included in this review, the studies had to be rated as ++ or +. A ++ rating meant that all or the majority of the criteria for quality were fulfilled, while a + rating meant that some of the important criteria were fulfilled. Examples of criteria that were required are; the cases and controls are taken from comparable populations; the same exclusion criteria are used for both cases and controls; comparison is made between participants and non-participants to establish their similarities or differences.

The first screening of titles and abstracts was made by one of the authors (JH). Abstracts which were of interest in the first screening were retrieved in full text copies. Two authors (JH and MH) independently examined the full text articles guided by the inclusion and exclusion criteria. Articles which passed the second step were examined a last time and data were extracted to a data abstraction form designed for the study.

### The inclusion criteria were as follows

- ST-elevation AMI
- A protocol-based clinical pre-hospital pathway meant that patients were identified in the pre-hospital field and were transported directly to a cath lab (primary analyses) or to a CCU or intensive coronary unit (ICU) (secondary analyses).
- In all the analyses, it should be possible to compare patients using a protocol-based pre-hospital clinical pathway with patients who were transported by the emergency medical service (EMS) to the ED.

### Exclusion criteria

Table 1 shows examples of studies [6-19] which, for various reasons, had exclusion criteria. The most common reasons for exclusions were a study design where the study did not compare direct cath-lab admission with ED admission, in-hospital pathways and studies in which the pathway group received thrombolysis instead of PCI.

**Table 1:** **Characteristics of excluded studies after abstract reading**

**Study**	**Reason for exclusion**
**Burns et al. 1989 [** 6 **]**	Prehospital pathway where the patients received thrombolysis
**Pell et al. 1992 [** 7 **]**	The study evaluated an in-hospital triage system
**Davis et al. 1996** [8]	A comparison between patients and physicians acceptance of risk
**Millar-Craig et al. 1997** [9]	Prehospital pathway where the patients received thrombolysis
**Prasad et al. 1997** [10]	Prehospital pathway where the patients received thrombolysis
**Thomas et al. 1997** [11]	Prehospital pathway where the patients received thrombolysis
**Sandler 1998** [12]	Prehospital pathway where the patients received thrombolysis
**Villiers et al. 2007** [13]	No control group
**Ostrzycki et al. 2008** [14]	The aim was to investigate time delay in treatment of STEMI patients in four different groups
**Zhang et al. 2009** [15]	The study evaluated the effect of in-hospital triage
**Cheung et al. 2010** [16]	The study evaluated the effect of in-hospital triage
**Sorensen et al 2011** [17]	The aim of the study was to bypass the local hospital and transfer patients direct to PCI center
**Abrahamyan et al.2012** [18]	The study evaluated the effect of in-hospital triage
**Alexandrescu et al. 2012** [19]	The study investigated the impact of inter-hospital transfer

### Outcomes and their definitions

Door-to-balloon time: the time when the patient arrived at hospital until the time when the balloon was inflated.

The time from symptom onset or first medical contact to balloon time: the time when reported symptoms started or when the EMS was called upon until balloon inflation.

Hospital mortality: the rate of death during first hospitalisation.

### Search methods

#### Electronic searches

Searches were made in the following databases in June 2012: *PubMed*, *Embase*, the *Cochrane Library* and *ProQuest Nursing & Allied Health Sources*. The searches were updated in January 2014, and then *ProQuest NAHS* was exchanged for *CINAHL*. The searches were restricted to studies in English, Swedish, Danish and Norwegian languages. The following words were used, with truncation and subject headings adapted to each database: (E*mergency department bypass OR Fast track OR Clinical Path OR Direct admission OR admitted direct OR Patient Admission OR Critical Pathways)* AND *(Myocardial Infarction OR Acute Coronary Syndrome OR heart infarction OR heart attack OR cardiac infarction)*. At a later stage the words STEMI OR ST-elevated myocardial infarction were added to the search, with no new relevant studies found. Full search strategies for all the databases are available upon request. In addition to the database searches we also scanned the reference lists of the included studies.

### Data synthesis

To estimate the average effect across the included studies, mean effects were calculated. For the dichotomous data, risk ratios with 95% confidence intervals were used. As we estimated that there was a possibility of variation in the true effect across the studies, we decided to use the “random effect” model [20]. Heterogeneity between the studies was tested by the standard chi-square test. For the continuous variables, it was not possible to perform a meta-analysis as all the included studies were presented as median and inter-quartile range. The continuous data are presented in narrative and tabular form. A significant effect of the pathway was defined by a P value less than 0.05. Analyses were performed using Review Manager (RevMan) [computer program], Version 5.1, Copenhagen: The Nordic Cochrane Centre, the Cochrane Collaboration, 2011.

## Results

### Description of studies

**Figure 1:**
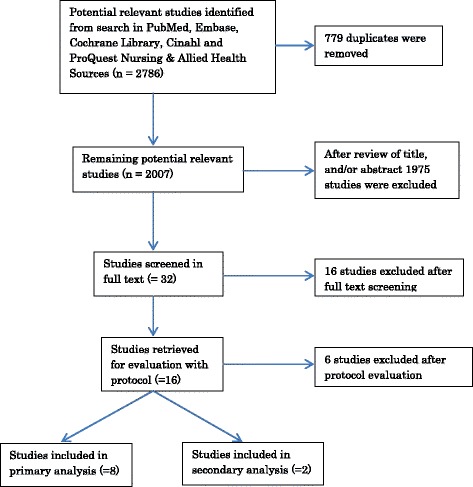
**Flow diagram of included and excluded studies.**

The search of the databases identified 2007unique citations (Figure 1). Of them, 1975 were excluded after the initial screening of titles and abstracts. Eight studies [21-28] met all the inclusion criteria for the primary analysis (Table 2). No included study had a randomised controlled design (RCT). The interventions in all the included studies were pre-hospital direct admission to a cath lab compared with the group which was admitted to the ED by the EMS and, after assessment by ED doctors, was transferred to a cath lab or CCU.

**Table 2:** **Characteristics of included studies**

**Study**	**Design**	**Duration**	**Diagnostic criteria**	**Intervention**	**Number of participants**	**Outcomes included in review**
**Carstensen et al. 2007** [21]	Prospective observational study	17 months	ST elevation>= 1 mm in >=2 contiguous leads or suspected new LBBB.	Direct admission to catheter lab.	Intervention: N=108	- Symptom onset to balloon time
					Control: N=193	
						- Door to balloon time
						- Hospital mortality
**Dorsch et al. 2008** [22]	Prospective observational study	14 months	Non traumatic chest pain within last 12 hours. ST elevation in 2 adjacent leads (>1 m V in leads I-III, aVL, and aVF, and >2 mV in V1-V6	Direct admission to catheter lab.	Intervention: N=172	- Door to balloon time
					Control: N=215	- Call to balloon time
						- 30 days mortality
**Bång et al. 2008** [23]	Prospective observational study	66.5 months	St elevation >= 1 mm in ECG leads I, II, III, AVF, V5 and V6 or >= 2mm in leads V1, V2, V3 and V4.	Direct admission to ICCU or catheter lab. The review used the catheter lab groups data	- Door- balloon time: Intervention N=115 control N=66	- Door-balloon time
						- From onset of symptoms to balloon time
					Symptoms - balloon time: Intervention N=115 control N=66	
**Grosgurin et al. 2010** [24]	Before and after	24 months	St elevation >=1 mm in 2 or more contiguous limb leads or ST elevation >= 2 mm in 2 or more contiguous precordial leads, or new LBBB	Direct admission to catheter lab.	Intervention: N=119	- Door to balloon time
					Control: N=77	
**Majumder et al. 2011** [25]	Retrospective observational study	31 months	Suspicion of STEMI not specified	Direct admission to catheter lab.	Intervention: N=200	- Door to balloon time
					Control: N=161	- Call to balloon time
**Cheskes et al. 2011** [26]	Before and after	24 months	ST elevation >1mm in at least 2 contiguous limb leads or 2 mm in at least 2 contiguous precordial leads or LBBB	Direct admission to catheter lab.	Intervention N=80 Control N=95	- EMS contact to balloon time
**Bagai et al. 2013a** [27]	Retrospective observational study	42 months	ST segment elevation >=1 mm in >= 2 contiguous ECG leads or LBBB or isolated posterior infarction	Direct admission to catheter lab.	Intervention N=1316 and control N=11265	- First medical contact to balloon time
						- Hospital mortality
**Bagai et al. 2013b** [28]	Retrospective observational study	18 months	ST segment elevation >=1 mm in >= 2 contiguous ECG leads or LBBB or isolated posterior infarction	Direct admission to catheter lab.	Intervention N=286 and control N=1401	- First medical contact to balloon time

In the included studies “door to balloon time” is defined as the time from first hospital door to balloon inflated. In this review “symptoms onset to balloon time” is equated to “first medical contact to balloon time”, “call to balloon time” and “EMS contact to balloon time”.

Direct admission to catheter laboratory vs ED admission.

Two studies were included in the secondary analysis [29,30], where the pathway group was admitted to a CCU.

### Primary analyses

#### Door-to-balloon time

*In five of the included studies* [21-25]*, the door-to-balloon time was reported. The median delay was significantly shorter in the intervention group in all five studies. The difference in median delay varied between 16 minutes and 47 minutes. Only in one* [21] *of the five studies was the difference below 25 minutes (Table**3**).*

**Table 3:** **Direct admission to catheter laboratory vs ED admission**

**Study ID**	**Number of participants**	**Time; pathway group (minutes)**	**Time; control group (minutes)**	**P value**
**Carstensen et al. 2007** [21]	P N=108,	MD=34 (IQR 27-48)	MD=50 (IQR 34-85)	P<0.001
	C N=193			
**Bång et al. 2008** [23]	P N=115,	MD=72 (IQR NR)	MD=97 (IQR NR)	P<0.001
	C N=66			
**Dorsch et al. 2008** [22]	P N=172	MD=58 (IQR NR)	MD=105 (IQR NR)	P<0.001
	C N=215			
**Grosgurin et al. 2010** [24]	P N=119	MD=71 (IQR 46-103)	MD=109 (IQR 74,5-149,5)	P<0.001
	C N=77			
**Majumder et al. 2011** [25]	P N=200	MD=39 (IQR 26-53)	MD=82 (IQR 49-120)	P<0.001
	C N=161			

N= Numbers.

P= Pathway.

C= Control.

MD= Median.

IQR= Interquartile range.

NR= Not reported.

Door to balloon time.

#### Onset of symptoms or first medical contact to balloon time

*In all seven included studies* [21-23,25-28]*, the time from symptom onset or first medical contact to balloon time was significantly shorter in the intervention group. The difference in median delay varied between 15 minutes and one hour and 35 minutes. Only in three* [25,27,28] *of the seven studies was the difference in median delay lower than 25 minutes (Table**4**).*

**Table 4:** **Direct admission to catheter laboratory vs ED admission**

**Study ID**	**Number of participants**	**Time; pathway group (minutes)**	**Time; control group (minutes)**	**P value**
**Carstensen et al. 2007** [21]	P N=108,	MD=154 (IQR 120-233)	MD= 249 (IQR 184-405)	P<0.001
	C N=193			
**Bång et al. 2008** [23]	P N=115,	MD=184	MD=238	P<0.02
	C N=66	(IQR NR)	(IQR NR)	
**Dorsch et al. 2008** [22]	P N=172	MD=105	MD=143	P<0.001
	C N=215	(IQR NR)	(IQR NR)	
**Majumder et al. 2011** [25]	P N=200	MD=106 (IQR 91-132)	MD=130 (IQR 103-164)	p<0.005
	C N=161			
**Cheskes et al. 2011** [26]	P N=80	MD=70 (IQR 24)	MD=107 (IQR 30)	P<0.001
	C N=95			
**Bagai et al. 2013a** [27]	P N=1316	MD=68 (IQR 54-85)	MD=88 (IQR 73-106)	P<0.001
	C N=11265			
**Bagai et al. 2013b** [28]	P N=28	MD=75 (IQR 59-93)	MD=90 (IQR 76-109)	P<0.001
	C N=1401			

N= Numbers.

P= Pathway.

C= Control.

MD= Median.

IQR= Interquartile range.

NR= Not reported.

Symptom onset to balloon time.

#### Hospital mortality

*Only two studies* [21,27] *describe hospital mortality. When combined, the risk of death was reduced by 37% (Additional file**1**).*

### Secondary analyses

#### Door-to-balloon time

*In two studies* [29,30]*, the median delay was significantly shorter in the intervention group (direct admission to CCU or ICU), with a shortening of the median delay of 24 min (p = <0.01)* [29] *and 34 min (p = <0.002)* [30] *respectively.*

## Discussion

This study, which is based on a systematic review of the available literature, suggests that a protocol-based pre-hospital clinical pathway with direct admission to a cath lab, thereby bypassing the ED, shortens delay to revascularisation and thereby improves outcome in STEMI.

It is important to remember that systematic reviews including non-randomised trials can be biased in terms of both under and over-estimates of treatment effects. The largest bias in non-randomised trials is selection bias [31].

It has been proposed that the most adequate estimate of delay to revascularisation in AMI is the delay from calling for the EMS until the start of treatment. This is called system delay [32].

We found that “door to balloon” and symptom or first medical contact to balloon was reduced by a time that varied from about 15 minutes to about one and a half hours. An overall mean reduction of about 30-40 minutes is therefore probably a realistic assumption.

What survival benefit is to be expected from such a relatively modest reduction in delay to revascularisation? The association between system delay and mortality, as well as the development of heart failure, was most clearly addressed by Therkelsen et al. [32]. They showed that, when adjusting for a variety of confounders, for each hour of increase in system delay, the risk of death and the risk of complications, defined as heart failure, increased by 10% during a three-year follow-up [33].

Our results, suggesting that the introduction of a protocol-based pre-hospital clinical pathway could be associated with a reduction in hospital mortality of about 30-40%, are more optimistic and deserve consideration.

As neither of the two studies referred to was a randomised clinical trial, the risk of selection bias is obvious.

1. The patient perspective: in all probability, health-care providers (HCP) tended to admit younger patients and patients with less comorbidity directly to a cath lab as compared with those who were transported to the ED. Most probably, patients with the most marked ST elevations were more frequently admitted directly to a cath lab.
2. The time perspective: in all probability, during the time, at weekends and during holiday months, the facilities for direct admission were inferior.
3. The HCP perspective: a more accurate pre-hospital diagnosis, greater use of aspirin and a more active approach to pain relief in the pre-hospital setting might be associated with direct admission to a cath lab.
4. The in-hospital treatment perspective: patients who were directly admitted to a cath lab most probably received anti-ischemic and anti-thrombotic medication more rapidly than those who were admitted to an ED.

The implications of these limitations are that the interpretation of data on clinical benefit must be careful and, without any randomised study, there is no clear evidence.

In the secondary analyses, we evaluated the impact of direct admission to an ICU/CCU. This was also associated with a significant reduction in door-to-balloon time. With the introduction of a pathway like this, patients can be transferred directly from a CCU to a cath lab.

### Clinical implications

A protocol-based pre-hospital clinical pathway in ST-elevation myocardial infarction with direct admission to a cath lab can be expected to reduce the delay to revascularisation by about 30-40 minutes. This can be expected to reduce mortality. The extent of this mortality reduction is probably over-emphasised in this meta-analysis, due to selection bias. Our analysis suggests a reduction in hospital mortality in the range of 30-40%. Due to the relatively small number of cases, the confidence limits were wide. When relating our findings on door-to-balloon time to possible clinical achievements, it might be realistic to assume a less marked mortality reduction.

From an ethical perspective, it appears questionable that any RCT comparing direct admission with transport to the ED in ST-elevation AMI will ever be performed. Our interpretation is that direct admission to a cath lab should be recommended in ST-elevation AMI, despite the lack of evidence in terms of clinical benefit.

## Conclusion

The present systematic review of available studies of the impact of a protocol-based pre-hospital clinical pathway with direct admission to a cath lab as compared with the standard transport to the ED in ST-elevation AMI suggests the following. The delay to the start of revascularisation will be reduced. The clinical benefit is not clearly evidence based. However, the documented association between system delay and outcome defends the use of the pathway.

## Additional file

Additional file 1:
**Direct admission to catheter laboratory vs ED admission.**

## References

[1] Terkelsen CJ, Jensen LO, Tilsted HH, Thaysen P, Ravkilde J, Johnsen SP, Trautner S, Andersen HR, Thuesen L, Lassen JF (2011). Primary percutaneous coronary intervention as a national reperfusion strategy in patients with ST-segment elevation myocardial infarction. Circulation.

[2] Fibrinolytic Therapy Trialists’ (FTT) Collaborative Group (1994). Indications for fibrinolytic therapy in suspected acute myocardial infarction: collaborative overview of early mortality and major morbidity results from all randomised trials of more than 1000 patients. Lancet.

[3] Morrison LJ, Verbeek PR, McDonald AC, Sawadsky BV, Cook DJ (2000). Mortality and prehospital thrombolysis for acute myocardial infarction: A meta-analysis. JAMA.

[4] Scottish Intercollegiate Guidelines Network: **SIGN 50: A guideline developer's handbook** [http://www.sign.ac.uk/]

[5] Deeks J, Dinnes J, D’Amico R, Sowden A, Sakarovitch C, Song F, Petticrew M, Altman DG (2003). Evaluating non-randomised intervention studies. Health Technol Assess.

[6] Burns JM, Hogg KJ, Rae AP, Hillis WS, Dunn FG (1989). Impact of a policy of direct admission to a coronary care unit on use of thrombolytic treatment. Br Heart J.

[7] Pell AC, Miller HC, Robertson CE, Fox KA (1992). Effect of “fast track” admission for acute myocardial infarction on delay to thrombolysis. BMJ.

[8] Davis MA, Keerbs A, Hoffman JR, Baraff LJ (1996). Admission decisions in emergency department chest pain patients at Low risk for myocardial infarction: patient versus physician preferences. Ann Emerg Med.

[9] Millar-Craig MW, Joy AV, Adamowicz M, Furber R, Thomas B (1997). Reduction in treatment delay by paramedic ECG diagnosis of myocardial infarction with direct CCU admission. Heart.

[10] Prasad N, Wright A, Hogg KJ, Dunn FG (1997). Direct admission to the coronary care unit by the ambulance service for patients with suspected myocardial infarction. Heart.

[11] Thomas D, Cooper L, Cooper J, Taylor D, Robb A (1997). Direct fast track admission to a coronary care unit. J R Coll Physicians Lond.

[12] Sandler DA (1998). Paramedic direct admission of heart-attack patients to a coronary-care unit. Lancet.

[13] de Villiers JS, Anderson T, McMeekin JD, Leung RCM, Traboulsi M (2007). Expedited transfer for primary percutaneous coronary intervention: a program evaluation. Can Med Assoc J.

[14] Ostrzycki C, Borowiec-Kocańda A, Zera T, Pieńkowska K, Drop-Dzwonkowska D (2008). Pre-hospital delay of treatment in patients with ST segment elevation myocardial infarction undergoing primary percutaneous coronary intervention: Experience of cardiac centre located in the vicinity of the centre of Warsaw. Kardiol Pol.

[15] Zhang FH, Zhang JS, Shen WF, Zhang RY, Qiu JP, Jin HG, Zhang JF, Wang XL, Jiang L, Liao ML, Hu J (2009). Impact of different clinical pathways on outcomes of patients with acute ST-segment elevation myocardial infarction undergoing primary percutaneous coronary intervention: The RAPID-AMI study. Chin Med J (Engl).

[16] Gary SH, Cheung KT, Lau CC, Chan HL, Chau CH, Wu KL, Cheung CY, Choi MC, Tse TS, Chan KK, Li SK (2010). Primary percutaneous coronary intervention for ST elevation myocardial infarction: performance with focus on timeliness of treatment. Hong Kong Med J.

[17] Sørensen JT, Terkelsen CJ, Nørgaard BL, Trautner S, Hansen TM, Bøtker HE, Lassen JF, Andersen HR (2011). Urban and rural implementation of pre-hospital diagnosis and direct referral for primary percutaneous coronary intervention in patients with acute ST-elevation myocardial infarction. Eur Heart J.

[18] Abrahamyan L, Austin PC, Donovan LR, Tu JV (2012). Standard admission orders can improve the management of acute myocardial infarction. International J Qual Health Care.

[19] Alexandrescu R, Bottle A, Jarman B, Aylin P (2012). Impact of transfer for angioplasty and distance on AMI in-hospital mortality. Acute Card Care.

[20] Cooper HM, Hedges LV, Valentine JC (2009). The handbook of research synthesis and meta-analysis.

[21] Carstensen S, Nelson GCI, Hansen PS, Macken L, Irons S, Flynn M, Kovoor P, Soo Hoo SY, Ward MR, Rasmussen HH (2007). Field triage to primary angioplasty combined with emergency department bypass reduces treatment delays and is associated with improved outcome. Eur Heart J.

[22] Dorsch MF, Greenwood JP, Priestley C, Somers K, Hague C, Blaxill JM, Wheatcroft SB, Mackintosh AF, McLenachan JM, Blackman DJ (2008). Direct ambulance admission to the cardiac catheterization laboratory significantly reduces door-to-balloon times in primary percutaneous coronary intervention. Am Heart J.

[23] Bång A, Grip L, Herlitz J, Kihlgren S, Karlsson T, Caidahl K, Hartford M (2008). Lower mortality after prehospital recognition and treatment followed by fast tracking to coronary care compared with admittance via emergency department in patients with ST-elevation myocardial infarction. Int J Cardiol.

[24] Grosgurin O, Plojoux J, Keller P-F, Niquille M, N’Koulou R, Mach F, Sarasin FP, Rutschmann OT (2010). Prehospital emergency physician activation of interventional cardiology team reduces door-to-balloon time in ST-elevation myocardial infarction. Swiss Med Wkly.

[25] Majumder B, Mavroudis C, Smith C, Coghlan JG, Shiu M, Rakhit RD (2012). Superior outcome with direct catheter laboratory access vs ED-activated primary percutaneous coronary intervention. Am J Emerg Med.

[26] Cheskes S, Turner L, Foggett R, Huiskamp M, Popov D, Thomson S, Sage G, Watson R, Verbeek R (2011). Paramedic contact to balloon in less than 90 minutes: a successful strategy for St-segment elevation myocardial infarction bypass to primary percutaneous coronary intervention in a Canadian emergency medical system. Prehosp Emerg Care.

[27] Bagai A, Jollis JG, Dauerman HL, Peng SA, Rokos IC, Bates ER, French WJ, Granger CB, Roe MT (2013). Emergency department bypass for ST-segment–elevation myocardial infarction patients identified with a prehospital electrocardiogram: a report from the American heart association mission: lifeline program. Circulation.

[28] Bagai AJ, Al-Khalidi HR, Munoz D, Monk L, Roettig ML, Corbett CC, Garvey JL, Wilson BH, Granger CB (2013). Bypassing the emergency department and time to reperfusion in patients with prehospital ST-segment-elevation findings from the reperfusion in acute myocardial infarction in Carolina emergency departments project. Circulation.

[29] Amit G, Cafri C, Gilutz H, Ilia R, Zahger D (2007). Benefit of direct ambulance to coronary care unit admission of acute myocardial infarction patients undergoing primary percutanoues intervention. Int J Cardiol.

[30] Lubovich A, Hamood H, Behar S, Rosenschein U (2011). Bypassing the emergency room to reduce door-to-balloon time and improve outcomes of patients with ST elevation myocardial infarction: the acute coronary syndrome Israeli survey experience. Isr Med Assoc J.

[31] Deeks JJ, D’Amico R, Sakarovitch C, Altman DG: **How big are the biases associated with non-randomised designs used in evaluations of healthcare interventions? An empirical investigation.** In *3rd Symposium on Systematic Reviews: Beyond the Basics.* July 2000 In Oxford [Online]. Retrieved from Http://Www.Mrw.Interscience.Wiley.Com/Cochrane/Clcmr/Articles/Cmr-2973/Frame.Html

[32] Terkelsen CJ, Sørensen JT, Maeng M, Jensen LO, Tilsted H-H, Trautner S, Vach W, Johnsen SP, Thuesen L, Lassen JF (2010). System delay and mortality among patients with STEMI treated with primary percutaneous coronary intervention. JAMA.

[33] Terkelsen CJ, Jensen LO, Tilsted H-H, Trautner S, Johnsen SP, Vach W, Bøtker HE, Thuesen L, Lassen JF (2011). Health care system delay and heart failure in patients with ST-segment elevation myocardial infarction treated with primary percutaneous coronary intervention: follow-up of population-based medical registry data. Ann Intern Med.

