# Supplementary material for: The impact of direct admission to a catheterisation lab/CCU in patients with ST-elevation myocardial infarction on the delay to reperfusion and early risk of death: results of a systematic review including meta-analysis
Source: Scand J Trauma Resusc Emerg Med. 2014 Nov 25;22:67. doi: 10.1186/s13049-014-0067-x (PMC4258278; doi:10.1186/s13049-014-0067-x)
Supplement: Additional file 1: — Direct admission to catheter laboratory vs ED admission. [file 13049_2014_67_MOESM1_ESM.docx]

**Additional file 1: Direct admission to catheter laboratory vs ED admission**

| **Direct admission to catheter laboratory** | | | **Admission via ED** | | | **Risk Ratio** | **Risk Ratio** |
| --- | --- | --- | --- | --- | --- | --- | --- |
| Study or Subgroup | Events | Total | Events | Total | Weight | M-H, Fixed, 95% CI | M-H, Fixed, 95% CI |
| Bagai et al. 2013a | 36 | 1316 | 462 | 11265 | 90.6% | 0.67 [0.48, 0.93] | 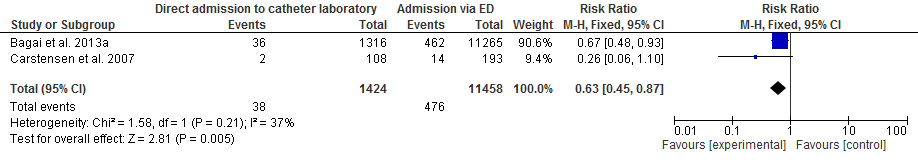 |
| Cartensen et al. 2007 | 2 | 108 | 14 | 193 | 9.4% | 0.26 [0.06, 1.10] |  |
| Total (95% CI) |  | 1424 |  | 11458 | 100.0% | 0.63 [0.45, 0.87] |  |
| Total events | 38 |  | 476 |  |  |  |  |
| Heterogeniety. Chi^2^=1.58, df=1 (P=0.21), I^2^=37% | | |  |  |  |  |  |
| Test for all effect: Z=2.81 (P=0.005) | | |  |  |  |  |  |

Hospital mortality
